# National HIV Testing Day — June 27, 2019

**DOI:** 10.15585/mmwr.mm6825a1

**Published:** 2019-06-28

National HIV Testing Day, observed each year on June 27, highlights the importance of testing in detecting, treating, and preventing human immunodeficiency virus (HIV) infection. Early diagnosis is critical to controlling HIV transmission in the United States (*1*). With the aim of reducing the number of new infections in the United States by 90% in 10 years, the Ending the HIV Epidemic initiative initially will focus on the 50 local jurisdictions where approximately half of diagnoses made in 2016 and 2017 were concentrated and in seven states with a disproportionate occurrence of HIV in rural areas (*2*). An analysis of 2016 and 2017 population-based survey data reported in this issue of *MMWR* found that overall, 38.9% of the U.S. population had ever tested for HIV infection, including 46.9% in the 50 local jurisdictions with the majority of diagnoses and 35.5% in the seven states with disproportionate occurrence of HIV in rural areas. To control HIV transmission, health care providers and public health practitioners need to develop HIV testing strategies to reach segments of the population that have never tested for HIV infection and offer at least annual testing of persons at risk for infection.

Additional information on National HIV Testing Day is available at https://www.cdc.gov/features/HIVtesting. Basic testing information for the public is available at https://www.cdc.gov/hiv/basics/testing.html. Additional information on HIV testing for health professionals is available at https://www.cdc.gov/hiv/testing. CDC’s guidelines for HIV testing of serum and plasma specimens are available at https://www.cdc.gov/hiv/guidelines/testing.html.
